# Microalgae Hybrid Biosystem for Enhanced Oral Delivery of Rifaximin in Hepatic Encephalopathy Treatment

**DOI:** 10.1002/advs.76443

**Published:** 2026-07-31

**Authors:** Kaiyue Wang, Xueting Huang, Lingxiao Yang, Jin Liu, Lei Lei, Shaomin Zou, Kai Wang, Min Zhou, Zhe Tang

**Affiliations:** ^1^ Department of Surgery and International School of Medicine International Institutes of Medicine the Fourth Affiliated Hospital of School of Medicine Zhejiang University Yiwu China; ^2^ Department of Radiology Sir Run Run Shaw Hospital Zhejiang University School of Medicine Hangzhou China; ^3^ Department of Neurosurgery School of Medicine the First Affiliated Hospital Zhejiang University Hangzhou China; ^4^ Department of Respiratory and Critical Care Medicine and International School of Medicine International Institutes of Medicine the Fourth Affiliated Hospital of School of Medicine Zhejiang University Yiwu China; ^5^ Zhejiang University‐University of Edinburgh Institute (ZJU‐UoE Institute) Zhejiang University School of Medicine Zhejiang University Haining China; ^6^ Department of Surgery The Second Affiliated Hospital Zhejiang University School of Medicine Hangzhou China

**Keywords:** blood ammonia, drug delivery, hepatic encephalopathy, nanoparticle, rifaximin, spirulina platensis

## Abstract

Hepatic encephalopathy (HE) is a serious neuropsychiatric complication of acute or chronic liver failure. It occurred in approximately 40% of acute liver failure cases and affected 30%–45% of patients with chronic liver failure or decompensated cirrhosis. Hyperammonemia is widely recognized as a central pathogenic factor in the pathogenesis of HE. Rifaximin (RIF), a non‐absorbable oral antibiotic, was widely used to manage HE by modulating gut microbiota and reducing ammonia production. However, its clinical efficacy remained suboptimal due to poor aqueous solubility, limited dispersibility, and inadequate gastrointestinal retention. These limitations were further exacerbated in severe cases, owing to restricted oral dosing, recurrence associated with poor adherence, and potential risks of antimicrobial resistance and infection‐related adverse events. This study presented a microalgae‐nanoparticle hybrid system (SP@RIF) for drug delivery, in which RIF‐encapsulated nanoparticles (RIFnano) were electrostatically loaded onto the surface of *Spirulina platensis* (SP), aiming to improve the oral delivery and therapeutic efficiency in HE treatment. RIFnano exhibited significantly enhanced antibacterial activity compared to free RIF. SP@RIF demonstrated prolonged gastrointestinal retention and sustained drug release profiles. In mouse models of HE, oral administration of SP@RIF markedly reduced systemic ammonia levels and improved behavioral outcomes. Furthermore, SP@RIF enhanced intestinal barrier function, attenuated systemic inflammation and neuroinflammation, ameliorated cognitive impairments, and modulated the gut microbiota. Importantly, these therapeutic benefits were achieved without observable toxicity. These findings highlight SP@RIF as a potential therapeutic strategy for treating HE.

## Graphical abstract

The microalgae‐nanoparticle hybrid system with antibacterial activity can improve the oral delivery and therapeutic efficiency for hepatic encephalopathy treatment. Oral administration of the system can reduced systemic ammonia levels, enhanced intestinal barrier function, attenuated systemic and neuroinflammation, ameliorated cognitive impairments, and modulated the gut microbiota.

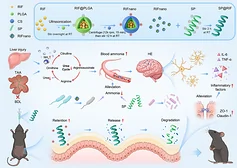

## 1 Introduction

Hepatic encephalopathy (HE) is a neurological complication resulting from acute or chronic liver failure and occurs in the context of portosystemic shunting [1]. It is characterized by a spectrum of cognitive and motor disturbances, including psychomotor dysfunction, memory impairment, prolonged reaction times, and disorientation. In severe cases, HE can progress to coma [2]. This condition reflects impaired brain function due to metabolic disturbances resulting from liver failure. The pathogenesis of HE is complex and remains incompletely understood. However, current evidence underscores the critical role of elevated blood ammonia levels in the development of HE [3]. Ammonia, a toxic byproduct of protein metabolism, is primarily produced by gut microbiota in the intestine [4, 5]. Under normal conditions, the liver detoxifies ammonia by converting it into urea through the urea cycle [6]. In cases of liver impairment, such as cirrhosis, this detoxification process is compromised, allowing ammonia to bypass hepatic metabolism and enter systemic circulation. The brain is particularly vulnerable to the toxic effects of ammonia accumulation [3]. Although astrocytes in the brain can detoxify ammonia by converting it into glutamine, this mechanism becomes overwhelmed in the context of hyperammonemia [7]. The excessive accumulation of glutamine leads to osmotic imbalance, cellular swelling, and disruption of brain energy metabolism, contributing to the clinical manifestations of HE [8, 9].

Therefore, many clinical therapies for HE target the gut to reduce the production and absorption of ammonia [10, 11]. These approaches typically involve a combination of pharmacological interventions, including lactulose, rifaximin (RIF), and probiotics, among other therapeutic approaches [12, 13]. Lactulose is considered to achieve ammonia‐lowering through two primary ways of reducing ammonia absorption and enhancing intestinal motility to increase the excretion of NH_3_, with risks of abdominal distension, flatulence, and diarrhea [14]. Alternatively, RIF, a broad‐spectrum antibiotic, reduces the population of ammonia‐producing bacteria in the gut to lower blood ammonia levels [15]. It also exerts anti‐inflammatory functions by mediating the regulation of microbial composition and modifying the gut, which can consequently improve brain function [16, 17]. Recent research has shown that RIF may also play a role in gut barrier repair by modulating gut‐derived neurotoxic substances and enhancing neuronal activity through the regulation of the gut‐brain axis. Specifically, RIF's ability to alter the intestinal microbiome can reduce endotoxemia and systemic inflammation, both of which are key factors in the pathogenesis of HE [18]. Additionally, RIF has low systemic bioavailability, which reduces the risk of antibiotic resistance, making it suitable for long‐term therapy [19]. Clinical evidence from randomized controlled trials has established that RIF has similar efficacy but better safety compared to lactulose or other antibiotics in the treatment of episodic HE [20, 21].

Despite its advantages, RIF has certain limitations. RIF typically exerts its therapeutic effects within the intestinal bile acid environment [22, 23]. This is attributed to the hydrophobic nature of bile acids, which facilitates a 70‐ to 120‐fold increase in the solubilization of RIF and significantly enhances its antibacterial activity [24]. This explains why RIF shows a dose‐dependent effect in the treatment of small intestinal bacterial overgrowth [25], while having a negligible effect in the colon [26]. Moreover, in patients with cirrhosis, reduced bile acid levels would further diminish its efficacy in the treatment of HE [27].

Hence, we propose a drug delivery strategy for RIF to enhance its overall therapeutic efficacy in HE, as shown in Figure 1. First, poly (lactic‐co‐glycolic acid) (PLGA) and chitosan (CS), both biodegradable and biocompatible polymers [28, 29], were used to encapsulate the RIF layer‐by‐layer, forming nanoparticles that enhance its dispersity and control its release. Second, *Spirulina platensis* (SP), a type of microalgae with a unique spiral shape, was employed here to load the RIF nanoparticles, enhancing their retention in both the small intestine and colon. In the past decade, SP has been proposed as a promising drug delivery carrier [30]. Its small size and adjustable surface functionality enable it to effectively load and release drugs [31], offering a more targeted therapeutic approach and thereby improving drug bioavailability and efficacy [32]. Furthermore, due to its rich bioactive components [33], SP not only provides excellent biocompatibility but also enhances the therapeutic effects of drugs through its natural antioxidant, anti‐inflammatory, and immune‐modulating properties [34, 35]. Through a synergistic effect, the combination of SP and RIF improved behavioral outcomes in HE mice, offering a promising therapeutic strategy with potential clinical applications for the treatment of HE.

**FIGURE 1:**
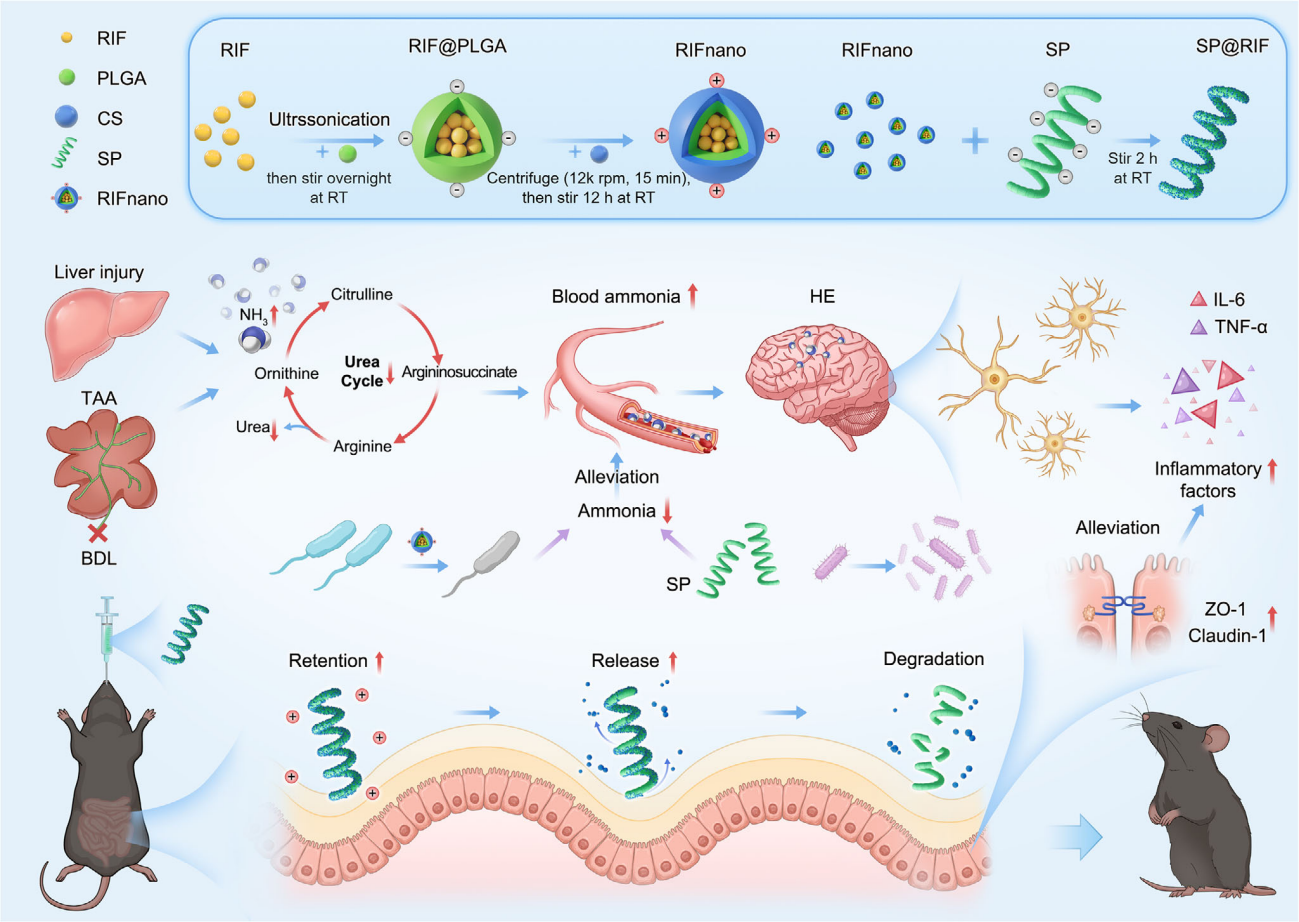
Schematic illustration of a microalgae‐nanoparticle hybrid system (SP@RIF) for HE treatment. RIFnano is loaded onto the surface of SP through electrostatic interactions. In mouse models of TAA‐ or BDL‐induced HE, oral administration of SP@RIF significantly reduced systemic ammonia levels and improved behavioral performance. Furthermore, SP@RIF enhanced intestinal barrier function, alleviated systemic and neuroinflammation, ameliorated cognitive impairment, and modulated the gut microbiota.

## 2 Results and Discussion

### 2.1 Preparation and Characterization of SP@RIF

To synthesize RIFnano, RIF was encapsulated within PLGA to form RIF‐loaded PLGA particles (RIF@PLGA), with a main size of 1000 nm in diameter (Figure 2E). To reduce the risk of exposure, the RIF@PLGA was designed to be slightly larger, thereby decreasing the likelihood of absorption by intestinal epithelial cells. Then, the RIF@PLGA was coated with another layer of quaternized CS, yielding RIF@PLGA@CS nanoparticles (RIFnano). Naturally, RIF's molecular structure, characterized by nonpolar hydrocarbon chains and aromatic rings (Figure 2B), imparts a hydrophobic nature, limiting its solubility in gut fluid. However, upon nanonization, RIFnano exhibited better dispersion in aqueous solutions than the free RIF powder (Figure 2C). Transmission electron microscopy (TEM) images reveal a spherical distribution of RIFnano particles (Figure 2D). Compared to RIF@PLGA, the RIFnano had an average diameter of approximately 2000 nm (Figure 2E) and exhibited a positively charged surface. The morphological stability of RIF nanoparticles under different storage conditions was examined by scanning electron microscopy (SEM). As shown in Figure S1, nanoparticles stored at 4°C retained a spherical shape and uniform size over 21 days, while those stored at room temperature and 37°C exhibited aggregation and surface irregularities from day 14. These findings suggested that lower storage temperatures better preserved the structural integrity of the nanoparticles. As shown in Figure 2G, the zeta potential of RIFnano increased from −5.8±0.25 mV (RIF@PLGA) to +40.43±0.56 mV, indicating the successful coating of quaternized CS.

**FIGURE 2:**
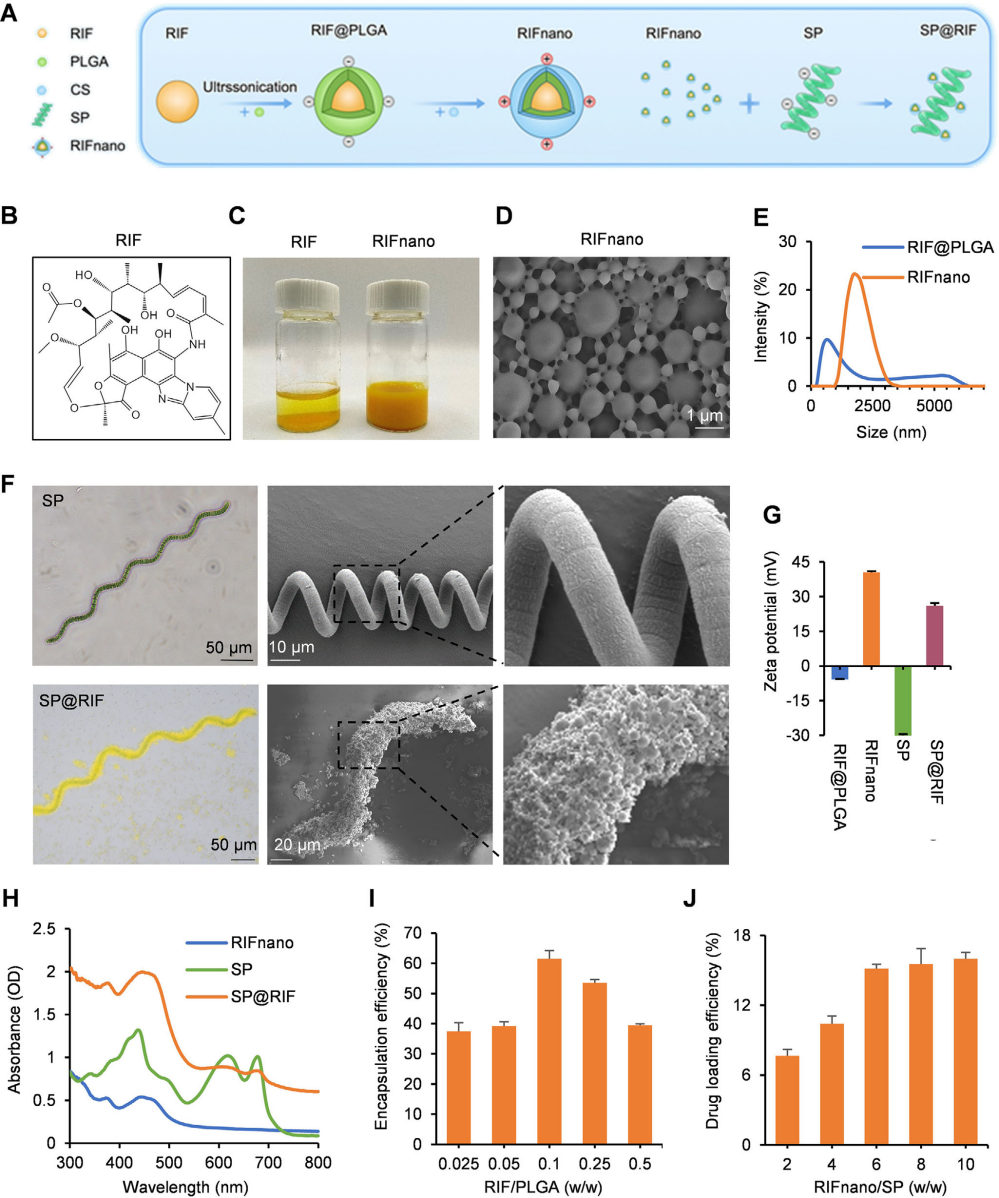
Synthesis and characterization of SP@RIF. (A) Synthesis of SP@RIF. RIF, rifaximin; PLGA, poly (lactic‐co‐glycolic acid); CS, chitosan; SP, *Spirulina platensis*. (B) The molecular formula of rifaximin. (C) Photographs of both RIF and RIFnano particles. (D) A representative SEM image of RIFnano particles. (E) Size distribution of RIF@PLGA and RIFnano particles. (F) Brightfield images (left) and the corresponding SEM images (middle) of SP and SP@RIF, respectively. Right, the surface of both SP and SP@RIF with dotted outlines was magnified. (G) Zeta potential of RIF@PLGA, RIF nanoparticles, SP, and SP@RIF (*n* = 3). (H) Ultraviolet spectra of RIFnano particles, SP, and SP@RIF. (I) Encapsulation efficiency under different weight ratios of RIF: PLGA (*n* = 3). (J) Drug loading efficiency under different weight ratios of RIFnano: SP (*n* = 3). Data are presented as means ± SD.

As shown in the design (Figure 2A), the positively charged RIFnano particles were easily loaded onto the surface of negatively charged SP through electrostatic interactions, forming an RIFnano‐loaded SP system (termed SP@RIF). The surface of SP typically carries negative charges, primarily due to the composition and chemical properties of its cell wall. During the preparation of SP@RIF, the zeta potential changed from −30.07±0.61 mV (SP) to +21±1.21 mV (SP@RIF) (Figure 2G). Microscopic observations revealed that SP changed significantly from green to yellow after being adorned with yellow RIFnano particles (Figure 2F). Additionally, SEM images showed that the smooth surface of SP was covered by massive particles, making it appear coarse after adhesion of the RIFnano. UV spectroscopic analysis also showed that SP@RIF presented the characteristic absorption peaks of RIFnano (Figure 2H). Taken together, these results further confirmed the successful loading of RIFnano onto the surface of SP. To investigate the encapsulation efficiency (EE) and drug loading efficiency (DLE) under varying mass ratios, we found that when the weight ratio of RIF to PLGA was 1:10, the EE reached its highest value of 61.45%±2.75% (Figure 2I). Similarly, when the weight ratio of RIFnano to SP was 1:6, the DLE reached a maximum of 15.98%±2.75% (Figure 2J).

### 2.2 Antibacterial Effect and Reduction of Ammonia In Vitro

To evaluate the extent to which RIFnano inhibits bacteria compared to free RIF, we performed a test as shown in Figure 3A. Gut microbiota dysbiosis, characterized by the expansion of potentially pathogenic bacteria such as *Enterobacteriaceae*, is a hallmark of HE and contributes to hyperammonemia [36]. *Escherichia coli* (*E. coli*), a predominant and well‐studied member of the *Enterobacteriaceae* family, was selected as a representative model organism to assess the antibacterial efficacy of RIFnano against this pathogenic group. *E. coli* was treated with equal amounts of free RIF and RIFnano. In contrast to both the free RIF and control groups, the LB medium treated with RIFnano appeared clearer and supported fewer colony formations on the LB plates (Figure 3B). Additionally, blank nano and SP, the constituent components of SP@RIF, were also tested for antibacterial activity, and the results showed negligible antibacterial effects (Figure S2). These results indicated that the growth of *E. coli* was largely suppressed by RIFnano. Quantitative analysis of colony formation revealed that RIFnano was approximately three orders of magnitude more effective as an antimicrobial agent than free RIF at the same dose (Figure 3C). RIF inhibits RNA synthesis by binding to the β subunit of bacterial RNA polymerase, preventing RNA synthesis and causing bacterial death. Consistently, Calcein‐AM/PI double staining of the *E. coli* showed that the control group was predominantly composed of live bacteria (green), but the RIFnano‐treated group consisted almost entirely of dead bacteria (red) (Figure 3D). Contrastingly, *E. coli* treated with free RIF remained largely viable. Compared to free RIF, RIFnano demonstrated superior antibacterial efficacy in vitro.

**FIGURE 3:**
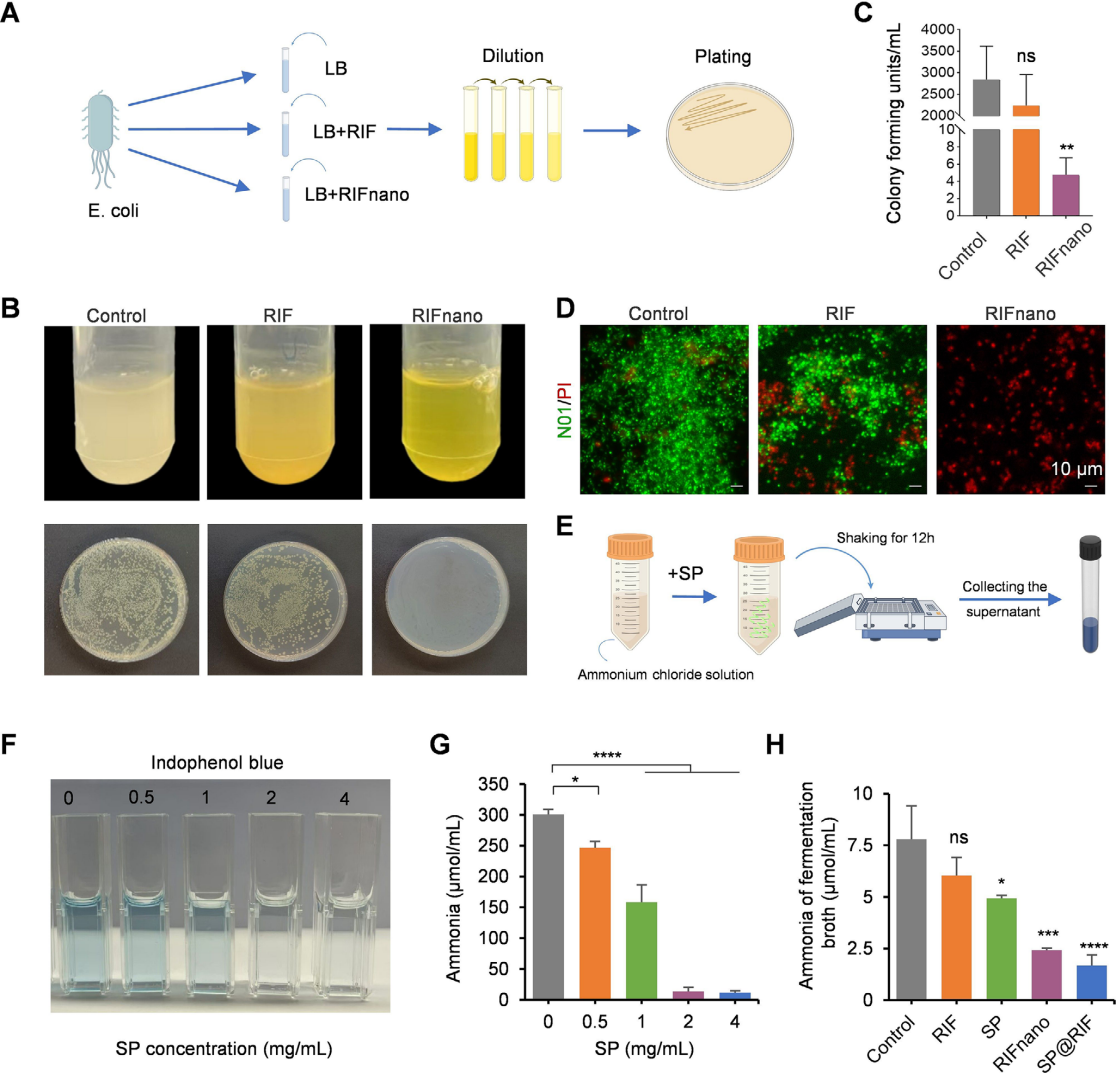
Ammonia‐lowering effect of SP@RIF. (A) Overview of the in vitro antibacterial experiment. (B) Images of *E. coli* in the tubes and the corresponding LB‐plate images of their colonies in different groups. (C) Quantifications of the colony‐forming units per milliliter from experiment B (*n* = 6). (D) N01/PI fluorescent images (green indicates living bacteria; red indicates dead bacteria) of the *E. coli* after 12 h with different treatments. (E) Experiment procedures for ammonia reduction using SP. (F) Photographs of the indophenol blue reaction used to measure ammonia concentration following treatment with different concentrations of SP. (G) Quantifications of ammonia levels from experiment E (*n* = 5). (H) Ammonia levels in the fermentation broth were quantified after 12 h for the indicated treatments (*n* = 5). Data are presented as means ± SD. *, p vs. Control group; ^*^, *p* < 0.05, ^**^, *p* < 0.01, ^***^, *p* < 0.001, ns, no significance.

Ammonia exists in the form of ammonia molecules (NH_3_) and ammonium ions (NH_4_^+^) in aqueous solution. It is widely used as a nitrogen source in many biological and chemical processes, especially in the metabolism of microalgae [37]. For environmental protection, microalgae have been used effectively to reduce ammonia concentrations in wastewater by absorbing and utilizing ammonia [38]. To investigate whether SP has a similar function, we performed the assay shown in Figure 3E. SP was added to the NH_4_Cl solution and incubated for 12 h on a shaker at room temperature. The supernatants were then collected to measure the ammonia concentration using the Indophenol Blue Colorimetric Method (IBCM). As shown in Figure 3F, the intensity of indophenol blue gradually decreased as the concentration of SP increased, indicating a reduction in ammonia content. Quantitative analysis of ammonia by UV–vis spectrometry revealed that SP was able to significantly absorb the ammonia and reduce its concentration in water (Figure 3G). To comprehensively evaluate the ammonia‐reducing effects of RIFnano's antibacterial activity and SP's ammonia‐absorbing effect, we added RIF, SP, RIFnano, and SP@RIF to LB medium with *E. coli*. After 12 h of incubation, the supernatants were collected to measure the ammonia concentration using IBCM. The results showed that the SP, RIFnano, and SP@RIF groups exhibited significant ammonia reduction, with the SP@RIF group showing the lowest ammonia concentration (Figure 3H), suggesting a synergistic effect of SP and SP@RIF in reducing ammonia levels.

### 2.3 Biological Distribution and Release Behaviors of SP@RIF In Vivo

PLGA and CS have both been widely used in drug delivery systems [39]. Their combination can enhance drug delivery effectiveness, stability, and other properties [40]. Differently, SP, as previously reported, helps prolong drug retention in the intestine through its distinctive filamentous helical structure. In this study, we performed a single oral administration of either free FITC and SP@RIF‐FITC in mice, followed by a series of gastrointestinal (GI) tract observations to assess retention. As expected, using a small animal in vivo imaging system, fluorescence from free FITC was observed within 2 h post‐gavage, whereas the SP@RIF‐FITC group showed sustained fluorescence for up to 8 h (Figure 4A). This significant difference indicated that SP@RIF‐FITC effectively prolonged the retention of the drug. Quantitative analysis showed that the fluorescence intensity of FITC in the SP@RIF‐FITC group was significantly higher than in the free FITC group (Figure 4B). Correspondingly, immunostaining of intestinal sections showed an in situ FITC signal in the colon in the SP@RIF‐FITC group, but not in the free FITC group, 4 h post‐gavage (Figure 4C). Thus, the SP@RIF system was able to extend the duration of the drug's presence in the gut, thereby improving bioavailability.

**FIGURE 4:**
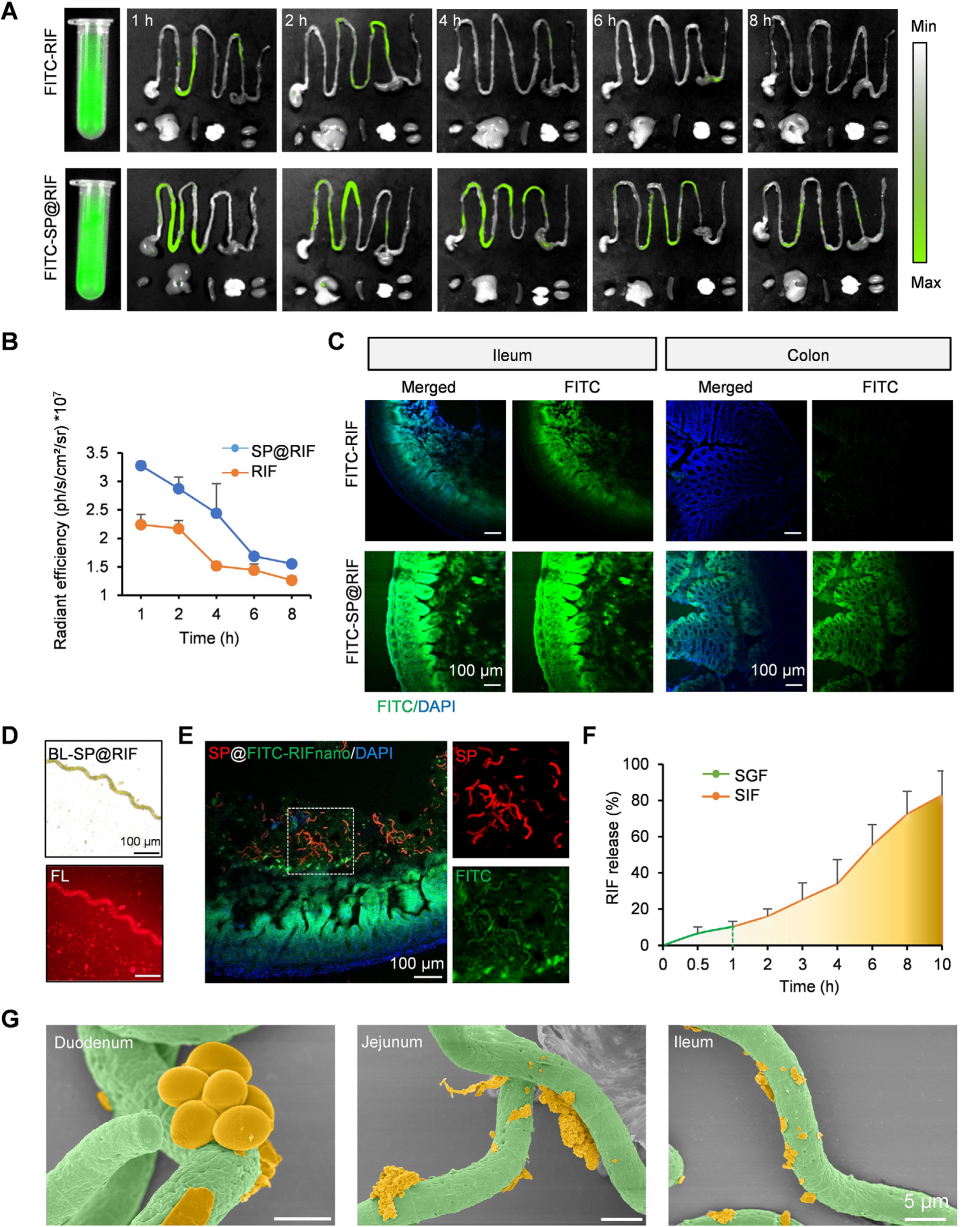
Biodistribution and drug release of SP@RIF. (A) Left: fluorescence imaging for FITC‐RIF and FITC‐SP@RIF in the tubes. Right, FITC fluorescence imaging of major mouse organs (gastrointestinal tract, heart, liver, spleen, lung, and kidney) at the indicated chase times following oral administrations of FITC‐RIF and FITC‐SP@RIF, respectively. (B) Quantification of FITC fluorescence intensity in the entire intestine in the mice from experiment A (*n* = 3). (C) Representative images showing the distribution of FITC fluorescence in the ileum and colon collected 4 h after oral administrations of FITC‐RIF and FITC‐SP@RIF, respectively. (D) Representative bright‐field and fluorescent images of SP@RIF. (E) Representative images showing the distribution of SP, a component from FITC‐SP@RIF, in the mouse intestinal lumen. (F) Release curves of RIF from RIFnano in simulated gastric fluid (SGF) and simulated intestinal fluid (SIF) (*n* = 3). Data are presented as means ± SD. (G) Representative SEM images (pseudocolor) of SP@RIF collected from intestinal contents 4 h after gavage, including the duodenum, jejunum, and ileum, respectively.

Next, we examined the intestinal contents in situ to assess the extent of the degradation process. SP's fluorescence primarily originates from its photosynthetic pigment, phycocyanobilin, which emits a bright red fluorescence under a 633 nm laser (Figure 4D). As shown in Figure 4E, SP from the ileum 4 h post‐gavage appeared partly fragmented but maintained its basic helical shape and self‐fluorescence, suggesting the stability of SP as a carrier in the intestine. To evaluate the release efficiency, SP@RIF was incubated in simulated gastric fluid (SGF) for 1 h and then incubated in simulated intestinal fluid (SIF) for an additional 9 h in vitro, in accordance with SP@RIF‐FITC distribution in the GI tract. UV spectroscopic analysis of RIF absorbance showed that less than 10% of RIF was released from SP@RIF in SGF, followed by a relatively steady release in SIF, reaching about 80% over a span of 10 h (Figure 4F). Additionally, we collected the intestinal contents from the duodenum, jejunum, and ileum 4 h post‐gavage. Using SEM, we observed that the number of RIFnano particles adhered to the SP gradually decreased along the intestinal axis (Figure 4G), indicating the near‐total release of RIF. Overall, the SP@RIF system achieved the slow release of RIF in the intestine.

### 2.4 The Neuroprotection of SP@RIF in HE Induced by Thioacetamide

Thioacetamide (TAA) is a hepatotoxin that has frequently been used to induce acute liver failure, causing experimental HE [41]. In this study, mice received continuous intraperitoneal injections of TAA for three days to induce HE and were subsequently subdivided into subgroups to receive either saline, RIF, SP, or SP@RIF oral administration every day for two weeks (Figure 5A). As expected, one day post the last TAA injection, liver function was determined to be severely impaired, with hepatic enzymes alanine aminotransferase (ALT) and aspartate transaminase (AST) significantly elevated above normal levels in the blood (Figure S3). Meanwhile, liver slides stained with H&E revealed inflammatory infiltrates and necrosis, and Sirius Red staining showed an increase in collagen fibrosis distribution and invasion into the liver parenchyma (Figure 5B), both of which enhance the likelihood of NH_3_‐rich portal blood entering the systemic circulation without detoxification. Compared to the saline control, blood ammonia levels in the TAA group sharply increased threefold one day post the last TAA injection (Figure 5C).

**FIGURE 5:**
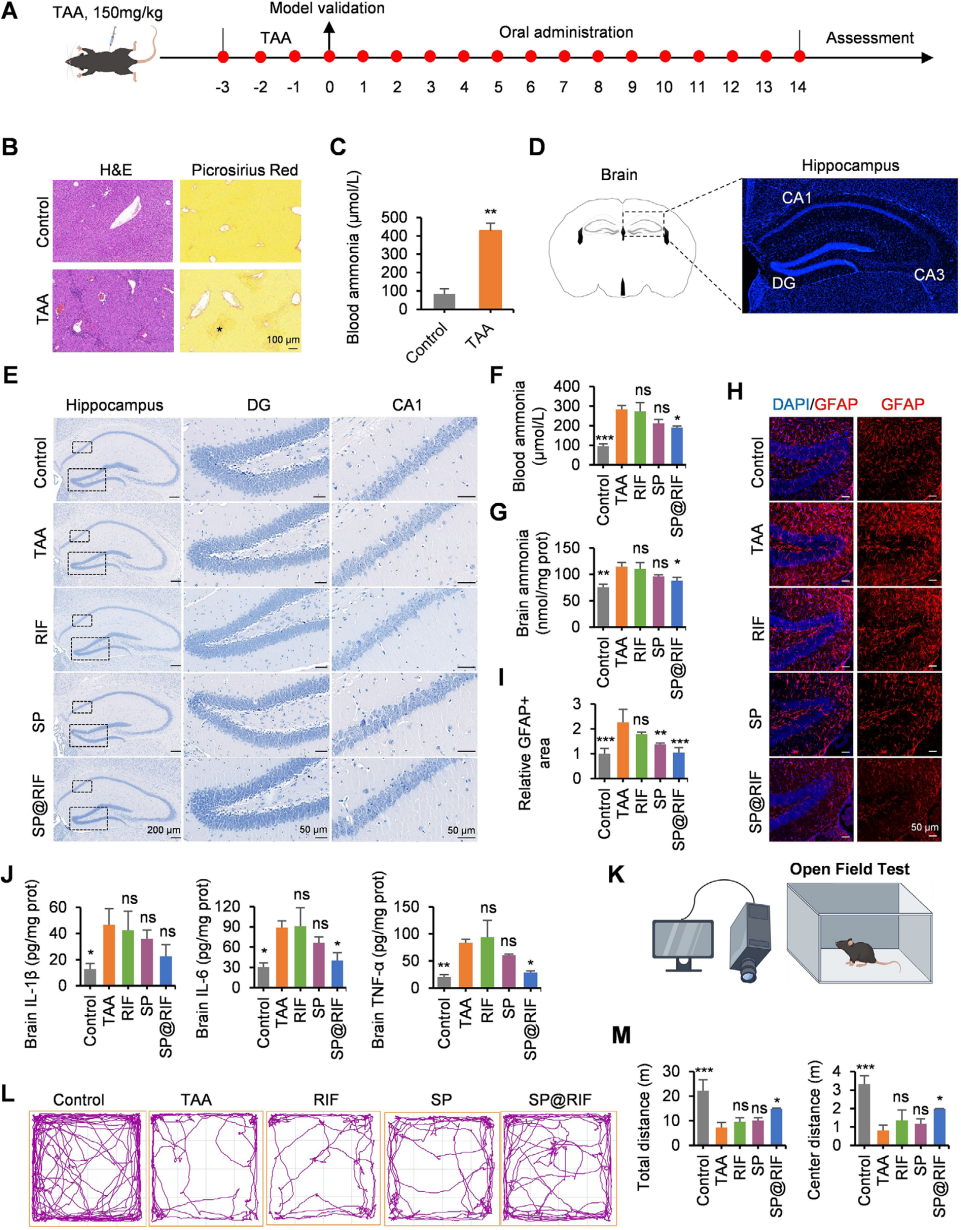
Brain protection of SP@RIF in TAA model mice. (A) Experiment overview. C57 mice were subjected to TAA injections to induce liver injury, followed by a recovery period of oral administration of RIF, SP, or SP@RIF as indicated. (B) H&E and picrosirius red staining of the liver in normal and TAA‐injection mice, with * indicating the typical areas of liver damage. (C) Blood ammonia levels in mice from experiment C (*n* = 5). (D) Diagram of the hippocampal region. (E) Representative histological images of the brain in each group. (F, G) Ammonia levels of blood (F) and brain (G) from each group were assessed. (H) Astrocytes (GFAP staining, red) in the dentate gyrus (DG) region of the hippocampus were assessed for inflammation. (I) Quantification of GFAP‐positive area in the DG region of the hippocampus (*n* = 3). (J) Levels of inflammatory factors, including IL ‐1β, IL‐6, and TNF‐α, in the mouse brain as shown (*n* = 5). (K) Mouse experiment schematic in the open field test (OFT). (L) Tracking plots show the position of the mice's central point for the entire duration of the OFT. (M) Quantifications of total distance and center distance traveled were calculated via Any‐Maze software (*n* = 5). Data are presented as means ± SD. *, p vs. TAA group; ^*^, *p* < 0.05, ^**^, *p* < 0.01, ^***^, *p* < 0.001, ^****^, *p* < 0.0001, ns, no significance.

The hippocampus, which plays a crucial role in memory, learning, and emotional regulation [42], is significantly affected in the context of HE [43, 44, 45]. In patients with HE, pathological examination of the hippocampus often reveals neuronal degeneration and apoptosis. Thus, we focused on the hippocampus to assess the extent to which SP@RIF may contribute to neuroprotection (Figure 5D). The CA1 region of the hippocampus is particularly sensitive to damage [46], often showing significant neuronal injury and apoptosis. Nissl staining of the CA1 region showed a significant reduction in cell density in the TAA group, while this situation was significantly improved in the SP@RIF group (Figure 5E). In comparison, the RIF group showed no significant difference compared with the saline group, but the SP group showed a mild increase in cell density. Furthermore, we examined the pathological changes in the dentate gyrus (DG) granule cell layer of the hippocampus, since the granule cells are particularly sensitive to ammonia's neurotoxic effects. Similarly, Nissl staining of the granular layer in the DG showed more closely and tightly arranged cell bodies with SP@RIF treatment than other treatments in HE. These results indicated that SP@RIF treatment had effectively reduced neuronal death in the hippocampus in HE.

The damage observed in the hippocampus was suggested to be related to ammonia toxicity and alterations in the neural environment, especially in neuroinflammation [47]. In HE, we observed a decrease in both blood and brain ammonia levels in the SP@RIF group (Figure 5F,G), while treatment with saline, RIF, and SP in the TAA‐induced HE model displayed no significant difference. This would explain the reduced extent of neuronal death in the hippocampus with SP@RIF treatment. As mentioned earlier, astrocytes are a type of glial cell in the central nervous system (CNS) that perform various critical functions, including detoxifying ammonia and helping to maintain the health and stability of the nervous system. In HE, the number of astrocytes in the hippocampus, identified by glial fibrillary acidic protein (GFAP) immunostaining, significantly increased alongside elevated ammonia levels (Figure S4). Hypertrophy of their cell bodies indicated the occurrence of neuroinflammation. After 14 days of administration, when comparing the relative intensity of astrocytes in DG among the different groups, the RIF group showed no significant decrease compared to the saline control group. In contrast, both the SP and SP@RIF groups exhibited a marked reduction in astrocyte levels (Figure 5H,I), suggesting that neuroinflammation was alleviated. This observation aligns with previous findings that SP possesses anti‐inflammatory properties. Microglia, another type of immune cell in the CNS, respond quickly to acute liver injury [48]. Their activation typically occurs within hours and involves changes in their morphology and function, including the release of pro‐inflammatory and anti‐inflammatory mediators. At the beginning of TAA‐induced HE, the number of microglia in the CA1 region increased, as shown by immunostaining for Ionized Calcium‐Binding Adapter Molecule 1 (Iba‐1) (Figure S4). After 14 days of administration, there were no significant differences in the relative intensity of Iba‐1 expression among these groups (Figure S5). However, the expression levels of pro‐inflammatory cytokines in the brain, especially IL‐6 and TNF‐α, were significantly reduced following SP@RIF treatment, whereas no such reduction was observed in the other groups (Figure 5J). The lower level of neuroinflammation represented less damage occurring in the brain following SP@RIF treatment.

To further assess improvement in brain function brought by SP@RIF, we examined the behavioral performance of mice using the open field test (OFT) (Figure 5K). Analysis of the tracking plots revealed that mice treated with SP@RIF in the OFT traveled a greater total distance and center distance compared to those treated with saline, RIF, or SP (Figure 5L,M). This indicated higher locomotor activity and improved brain function. Consistent with the above findings, these results suggested that SP@RIF provides more effective neuroprotection than RIF against TAA‐induced experimental HE.

### 2.5 The Protective Effects of SP@RIF on the Liver Through the Gut‐Liver Axis

Growing evidence demonstrates that neuroinflammation in HE is often associated with systemic inflammatory responses [49]. Systemic inflammatory cytokines, including IL‐1β, IL‐6, and TNF‐α, were measured in the blood. Consistent with inflammatory effects in the brain, the SP@RIF group exhibited a significant reduction in blood IL‐6 and TNF‐α levels compared to the other groups, though the RIF group showed a lower level of IL‐1β than the SP@RIF group (Figure 6A). Additionally, we measured the liver disease‐related inflammatory markers IFN‐γ and CXCL1. The SP@RIF treatment significantly reduced the levels of both IFN‐γ and CXCL1 compared with the TAA group, and no significant difference was observed between the normal control and SP@RIF groups (Figure S6). Collectively, these results suggested that SP@RIF would contribute to reducing systemic inflammation levels.

**FIGURE 6:**
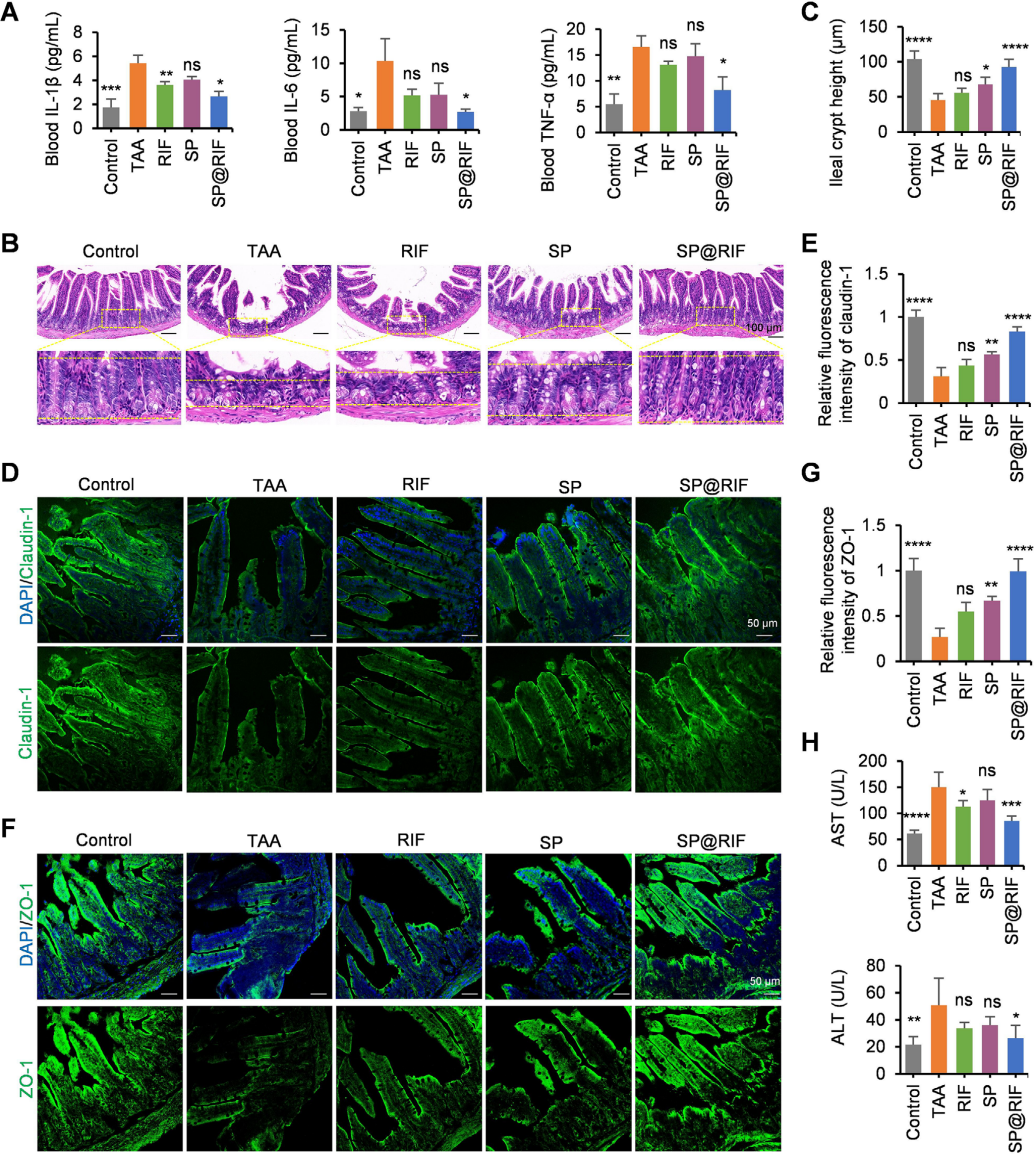
Systemic anti‐inflammation analysis and intestinal protection of SP@RIF. (A) Serum cytokines IL‐1β, IL‐6, and TNF‐α (H) in mice from each treatment group. (B) Representative images of the ileum. (C) Quantitative analysis of ileal crypt height (*n* = 3, 20 crypts per mouse). (D, F) Immunofluorescence staining for Claudin‐1 (D) and ZO‐1 (F) in the ileum. (E, G) Quantitative analysis of Claudin‐1 (E) and ZO‐1 (G) expression (*n* = 3). (H) Levels of AST and ALT in mice from each treatment group. Data are presented as means ± SD. *, p vs. TAA group; ^*^, *p* < 0.05, ^**^, *p* < 0.01, ^***^, *p* < 0.001, ^****^, *p* < 0.0001, ns, no significance.

However, RIF is a non‐systemic antibiotic that primarily acts on gut bacteria. The systemic inflammatory response induced by TAA can also affect the gut, leading to localized intestinal inflammation and impairing gut barrier function [50]. Therefore, we wondered whether SP@RIF affects the liver‐brain axis through its impact on the gut. Analysis of intestinal morphology via H&E staining showed that the crypt height in the ileum of SP@RIF‐treated mice remained relatively normal, similar to that of the normal control mice (Figure 6B,C). The SP group exhibited mild improvement in this aspect, while the RIF group showed less improvement and no significant change. Crypts are key structures that house intestinal stem cells and are crucial for regenerative capacity and maintaining the barrier [51]. The observed changes in crypt structure suggested that the intestine was damaged by the TAA modeling method but was revived by SP@RIF treatment. Furthermore, we measured the expression of tight junction proteins, including Claudin‐1 and ZO‐1, which contribute to the mechanical strength of the gut barrier [52]. Immunostaining revealed a disruption in the expression of Claudin‐1 and ZO‐1 in the TAA groups (Figure 6C,D), which would lead to increased intestinal permeability and aggravate liver injury through the gut‐liver axis. However, RIF treatment showed only marginal differences compared to the TAA group, whereas both SP and SP@RIF treatments led to increased expression of Claudin‐1 and ZO‐1 (Figure 6D,F). These results indicated that SP and SP@RIF help maintain intestinal barrier integrity, which may contribute to lowering systemic exposure to gut‐derived ammonia.

As expected, during the oral administration, the mice treated with RIF, SP, and SP@RIF exhibited more stable body weight than those treated with saline, particularly at 3 days post‐TAA injection (Figure S7). At 14 days post‐TAA injection, the levels of total bilirubin (TB), blood urea nitrogen (BUN), creatinine (CR), and direct bilirubin (DB) in the different groups approached normal ranges (Figure S8), implying that both liver and kidney functions were nearing normalcy. However, the hepatic enzymes ALT and AST in mice treated with SP@RIF showed the most significant decrease compared to other treatments in HE models (Figure 6G). This indicated that SP@RIF was effectively reducing liver injury and improving liver health, suggesting its potential role in liver protection through the gut‐liver axis.

### 2.6 The Neuroprotection of SP@RIF in HE Induced by Bile Duct Ligation

Bile duct ligation (BDL) in rodents is commonly used to induce chronic liver injury, involving surgical ligation of the bile duct, which leads to obstructive cholestasis [53]. Here, mice underwent either BDL surgery or a sham surgery involving laparotomy without BDL, followed by model validation 14 days post‐surgery (Figure 7A). The bile accumulation caused hepatocyte damage and inflammation (Figure S9A), ultimately resulting in liver failure, as previously reported. The levels of ALT and AST in the BDL group were approximately 2‐ to 3‐fold higher compared to the sham group, and the blood ammonia level in the BDL group increased by approximately onefold compared to the sham group (Figure S9B,C). However, the levels of TB and DB in the BDL group were hundreds of times higher than those in the sham group (Figure S9D). Notably, in the BDL group, the accumulation of bile acids, bilirubin, and other metabolic products in the liver led to more pronounced neuronal damage and the development of HE. Consistently, in the BDL group, Nissl staining of the hippocampus demonstrated a marked thinning of the granular layer in the DG region compared to the sham group (Figure S10), indicating a significant loss of neurons in the hippocampus and consequent impairment of brain function. These neuropathological changes in the BDL model were more pronounced than those in the TAA model.

**FIGURE 7:**
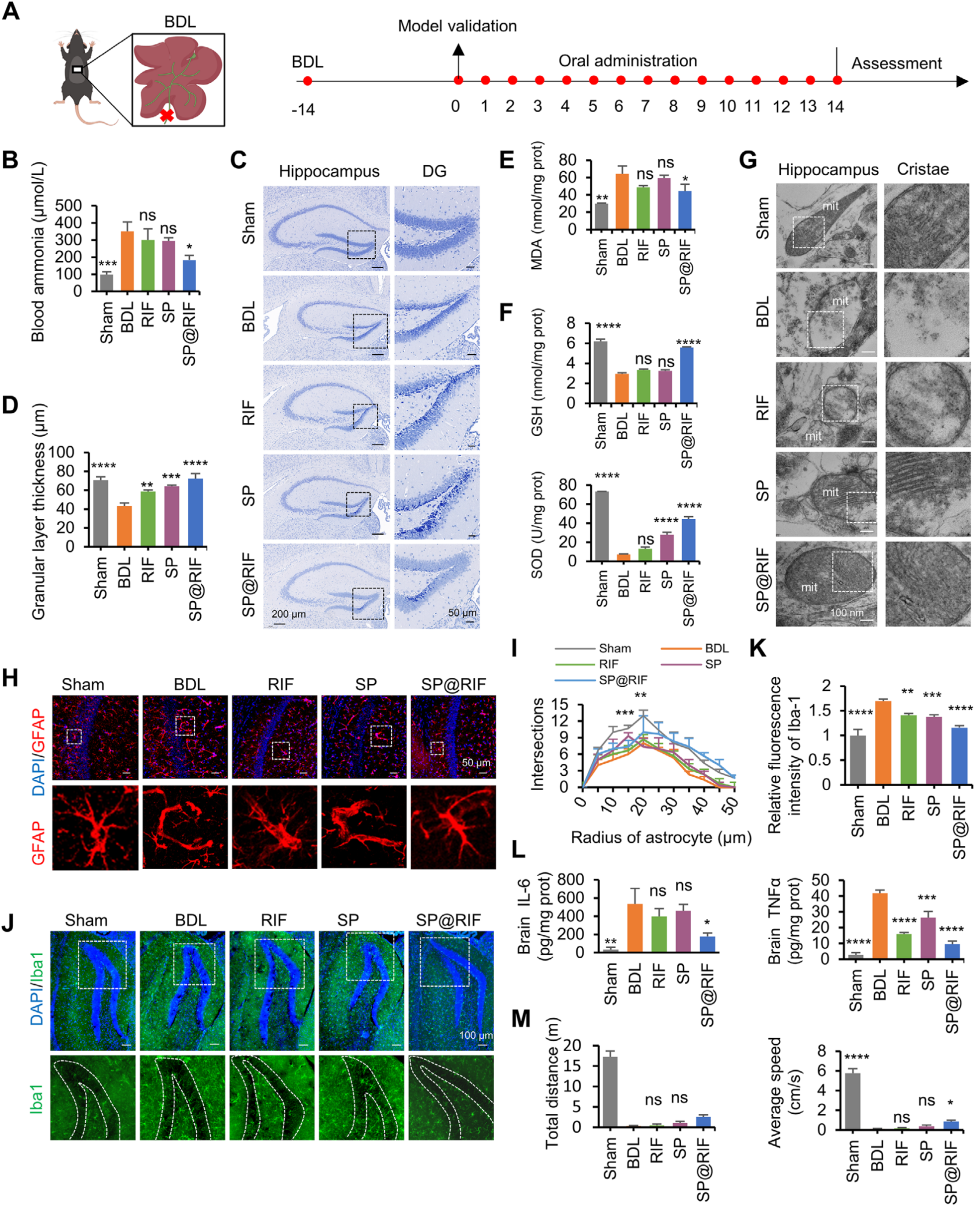
Effects of SP@RIF on brain protection in BDL model mice. (A) Experiment overview. C57 mice underwent BDL to induce liver injury, followed by a recovery period of oral administration of RIF, SP, or SP@RIF as indicated. (B) Blood and brain ammonia levels in mice from each group (*n* = 3). (C) Representative histological images of the brain in each group. (D) Quantification of brain granular layer thickness (*n* = 3). (E, F) Levels of oxidative stress markers, including MDA, GSH, and SOD in mice measured under various conditions (*n* = 3). (G) Representative TEM images of mitochondrial morphology in the brains of mice from each group, highlighting damaged mitochondrial cristae with white arrows. (H) Astrocyte staining with GFAP (red) in the CA1 region of the hippocampus. (I) Sholl analysis of astrocytes (*n* = 3). (J) Microglia staining with Iba‐1 (green) in the DG region of the hippocampus. (K) Quantification of Iba‐1 expression in the DG region of the hippocampus (*n* = 3). (L) Levels of IL‐6 and TNF‐α in the brain (*n* = 3). (M) Quantifications of total distance and average speed from the OFT after recovery treatments, analyzed using Any‐Maze software (*n* = 3). Data are presented as means ± SD. *, p vs. BDL group; ^*^, *p* < 0.05, ^**^, *p* < 0.01, ^***^, *p* < 0.001, ^****^, *p* < 0.0001, ns, no significance.

Subsequently, to evaluate the respective efficacies of RIF, SP, and SP@RIF, the mice in both the sham and BDL groups received oral treatments for an additional 14 days. At 28 days post‐surgery, the blood ammonia level in the BDL group was more than twice that of the sham group (Figure 7B). In the BDL model, SP@RIF treatment significantly decreased both blood and brain ammonia levels, whereas RIF and SP treatments showed less decrease and no significant changes. Pathological examination of the hippocampus revealed that the granular layer in the DG region was significantly thickened in all three treatment groups compared to the BDL group, with the SP@RIF group showing the most improvement (Figure 7C,D). This suggested that SP@RIF treatment reduced neuronal damage and death.

Given the toxicity of bile acids and bilirubin to the brain, we measured the level of oxidative stress. The marker of lipid peroxidation, malondialdehyde (MDA) [54], increased in the BDL group but decreased in the SP@RIF group (Figure 7E). Correspondingly, the levels of the antioxidant enzyme superoxide dismutase (SOD) and the antioxidant glutathione (GSH) were significantly increased in the SP@RIF group (Figure 7F). The level of SOD also increased in mice treated with SP, indicating the antioxidant effect of SP, as previously reported. Furthermore, using TEM, we observed that compared to the sham mice, the BDL mice displayed mitochondrial swelling and irregular or disappearing cristae in the hippocampus (Figure 7G). The SP@RIF treatment largely improved these mitochondrial abnormalities. Thus, these results suggest that SP@RIF treatment could reduce oxidative stress and protect neurons.

Additionally, we assessed the extent of neuroinflammation among these groups in the BDL model. Since the damage to the brain caused by BDL modeling was more severe than that caused by TAA modeling, the impact was more pronounced. In the BDL group, astrocytes exhibited phenotypic changes characterized by enlarged cell bodies and thicker, simplified processes compared to the sham group (Figure 7H). Complex branching in astrocytes commonly facilitates the efficient transport of nutrients and waste products between neurons and the blood–brain barrier [55]. In contrast, thicker and simplified branching patterns are often indicative of neuroinflammation and pathological conditions. Sholl analysis of astrocyte branching revealed a reduction in dendritic length and branching in the BDL group, while the SP@RIF group showed a higher number of concentric circle intersections compared to the RIF and SP groups (Figure 7I), indicating more complex dendritic branching in the SP@RIF group. Meanwhile, we observed that SP@RIF treatment significantly reduced the expression of Iba‐1 in the DG region compared to the BDL group (Figure 7J,K), suggesting a potential inhibitory effect on microglial activation. Quantitative analysis of pro‐inflammatory cytokines in the brain revealed that RIF, SP, and SP@RIF treatments significantly reduced TNF‐α expression, with the SP@RIF treatment showing the greatest decreases in the levels of both IL‐6 and TNF‐α (Figure 7L). These results suggest that SP@RIF treatment reduces inflammation in the brain, providing greater neuronal protection compared to other treatments.

To assess the neuroprotective effects of SP@RIF, mice were subjected to the OFT. Due to the irreversible and progressive liver damage caused by BDL, mice in the BDL group exhibited near‐total immobility, and neither SP nor RIF alone could effectively manage this condition. However, SP@RIF treatment significantly improved locomotor activity, as evidenced by increased total distance traveled and average speed based on the analysis of tracking plots (Figure 7M and Figure S11). In terms of overall behavioral outcomes, SP@RIF treatment had a limited effect on improving brain function in BDL‐induced HE.

### 2.7 Protective Effects of SP@RIF on the Gut in the BDL Model

As mentioned above, liver dysfunction in mice induced by BDL surgery was quite severe. A 70% mortality rate was observed in the BDL group, which was reduced to 20% with SP@RIF treatment and to 50% with SP treatment, 28 days post‐BDL surgery (Figure S12). The RIF treatment didn't provide any significant improvement. Liver function in the mice did not recover, as ALT, AST, TB, DB, BUN, and CR levels remained high and abnormal (Figure 8A–C) as long as the mice underwent BDL surgery. Only ALT levels were lower in the SP@RIF group, followed by the RIF group. These pathological states were further evidenced by liver slides stained with H&E (Figure 8E), which indicated that SP@RIF had no marked effect on liver protection in the BDL model.

**FIGURE 8:**
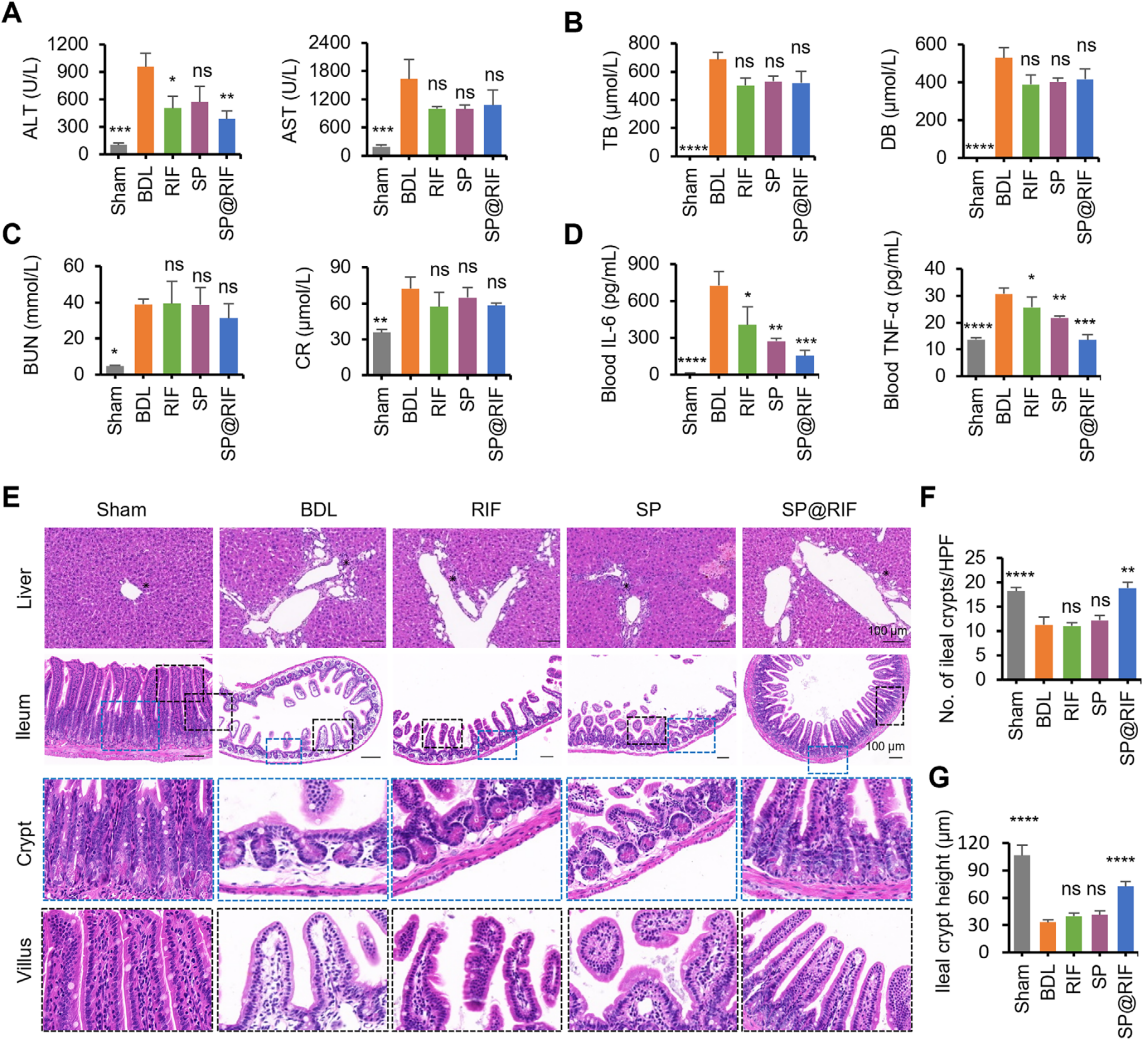
Systemic anti‐inflammation analysis and intestinal protection of SP@RIF in BDL model mice. (A, B) Hepatic enzyme (A) and bilirubin (B) levels in treated mice (*n* = 3). (C) BUN and CR levels in treated mice (*n* = 3). (D) Levels of pro‐inflammatory cytokines (IL‐6 and TNF‐α) in blood (*n* = 3). (E) Representative histological images of the liver and ileum in each group. (F) Number of ileal crypts per high‐power field (*n* = 3, 3 fields per mouse). (G) Quantitative measurement of ileal crypt height (*n* = 3, 20 crypts per mouse). Data are presented as means ± SD. Statistical significance: *, p vs. BDL group; ^*^, *p* < 0.05; ^**^, *p* < 0.01; ^***^, *p* < 0.001; ^****^, *p* < 0.0001; ns, no significance.

This liver damage swept across the whole body with systemic inflammation [56], leading to an increase in inflammatory cytokines, including IL‐6 and TNF‐α (Figure 8D). Among the treatments, SP@RIF had the most significant effect on reducing inflammatory factors, with SP showing a moderate effect and RIF having the weakest effect. Besides the liver damage, cholestasis caused by BDL also impaired gut function. After BDL surgery, bile could not drain into the duodenum, impairing the absorption of lipids by the intestinal epithelium and reducing the antibacterial efficacy of RIF. Moreover, bile acids possess antimicrobial properties, and their deficiency leads to dysbiosis, increasing the proportion of pathogenic bacteria and enhancing ammonia production [57]. In this context, histopathological analysis of the ileum revealed atrophy and disappearance of villi (Figure 8E), as well as a reduction in the height and number of crypts in the BDL group compared to the sham group (Figure 8F,G). This indicated severe disruption of the intestinal barrier in BDL‐operated mice. In contrast, only the SP@RIF group exhibited tightly arranged villi with significantly higher crypt height and number than the RIF and SP groups, indicating the protective effect of SP@RIF on the gut.

### 2.8 The Regulation of Gut Microbiota by SP@RIF

In this study, we aimed to reduce intestinal microbiota to decrease ammonia production, thereby reducing ammonia's neurotoxicity to the brain and facilitating recovery from HE. In BDL mice, the histopathological phenotypes of the intestine were significantly different between the RIF and SP@RIF treatments. Considering the close correlation between the gut and its luminal microbiota, we hypothesized that SP@RIF selectively reduced certain microbiota, thereby causing these observed differences. To address this question, we conducted 16S ribosomal RNA (rRNA) gene sequencing of fecal samples from mice in the RIF, SP, and SP@RIF groups following BDL surgery, in order to analyze shifts in the mouse intestinal microbiome.

Alpha diversity refers to the species richness and diversity within a locally uniform habitat [58]. The Chao1 index was used to characterize richness, and the Shannon index was employed to represent diversity in the 16S rRNA gene sequencing analysis. The median, as a statistical measure, provided a reference point for understanding how the Sham, SP, SP@RIF, and BDL experimental groups differed relative to a general baseline. As shown in Figure 9A, the mean values of the Chao1 index and Shannon index in the sham group were close to the median, representing the basal level of species richness and diversity in a healthy gut environment. However, due to the antimicrobial effect of RIF, the mean values of the Chao 1 index and Shannon index in the RIF and SP@RIF groups were lower than the median, particularly in the RIF group, which showed a significant decrease. Surprisingly, these values in the BDL‐induced HE mice were higher than the median levels, which may be caused by the loss of bile acids in the gut, as bile acids are known to have antimicrobial effects. To analyze the variance between these groups, the beta diversity index was used [59]. The principal coordinate analysis (PCoA) plot showed a relatively decentralized distribution of each group, demonstrating distinct differences in microbial composition across them (Figure 9B). Compared to the BDL group, the Jaccard distance was calculated to reveal the discrepancies between the BDL group and the other groups (Figure 9C) [60]. Among the groups, the RIF and SP groups exhibited microbial compositions closest to that of the BDL group. However, the SP@RIF group showed the greatest divergence from the BDL group. This suggested that SP@RIF had a significant effect on the richness and diversity of the gut microbiota in BDL mice.

**FIGURE 9:**
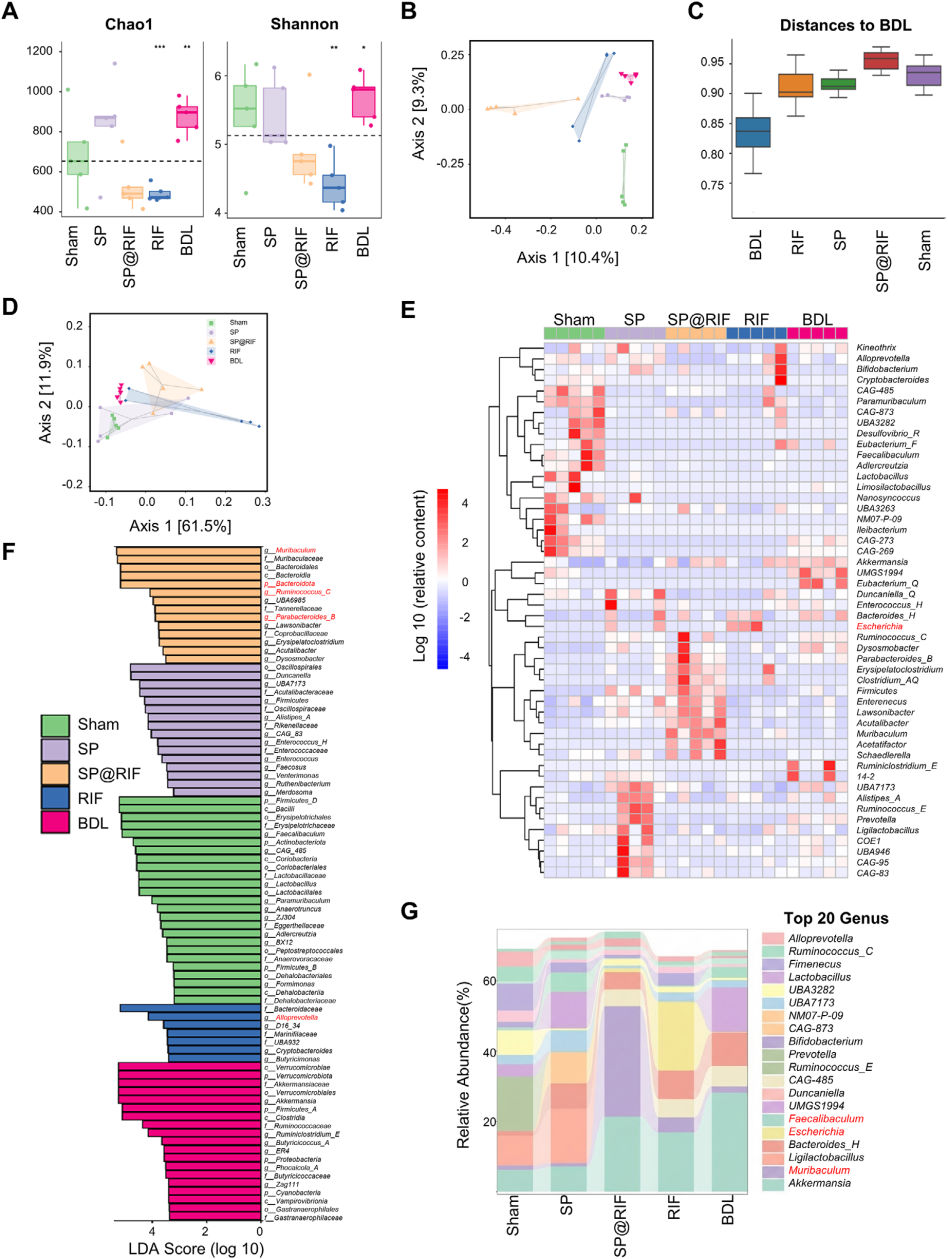
16S rRNA gene sequencing analysis of microbiota in BDL model mice. (A) Comparison of alpha diversity indices (Chao1 and Shannon) among groups. (B) Principal Coordinates Analysis (PCoA) plot based on Jaccard distance, illustrating microbial community differences. (C) Box plots with statistical tests for multiple group comparisons using distance matrix data. (D) PCoA plot based on Bray‐Curtis distance for beta diversity analysis. (E) Genus‐level heatmap showing species composition and clustering. (F) Microbial communities with a Linear Discriminant Analysis (LDA) score greater than 2 were analyzed using Linear Discriminant Analysis Effect Size (LEfSe). (G) Community bar plot displaying the relative abundance of microbiota at the genus level.

To explore functional differences between the samples, the Bray‐Curtis distance matrix was calculated, and PCoA analysis was performed (Figure 9D) [61]. A closer projection distance between samples on the coordinate axes indicated greater similarity in their functional composition. The results revealed that the SP and Sham groups were more similar, while the BDL and RIF groups showed relatively similar distances. However, the SP@RIF group was significantly clearly separated from the others, indicating a marked functional divergence in microbiota.

To further investigate the differences among the groups, a hierarchical clustering heatmap of genus‐level species composition was generated to highlight the relative abundance of distinct microbial populations in each sample across all groups (Figure 9E). Additionally, Linear Discriminant Analysis Effect Size (LEfSe) was performed to identify characteristic microbial communities (Figure 9F) [62]. Furthermore, the composition rates of the top 20 genera were calculated to visually illustrate the differences among these groups (Figure 9G and Figure S13). These results revealed that the species composition of the BDL and RIF groups was quite similar, while the microbial communities in the SP and Sham groups shared greater similarities. In the RIF group, the relative abundance of *Escherichia* was one of the highest among all groups. Clinical studies have indicated that severe dysbiosis in the gut microbiota of HE patients often leads to an overgrowth of pathogenic *E. coli* [63, 64]. Research has demonstrated that this overgrowth is linked to the production of lipopolysaccharides and ammonia [65]. In the BDL model, the absence of bile acids and the inability of RIF to effectively suppress *E. coli* growth may contribute to the unsatisfactory therapeutic outcomes in the treatment of HE. Additionally, the RIF group showed a higher abundance of *Alloprevotella* [66], which has been associated with intestinal villus damage.

In contrast to the BDL group, the SP@RIF group exhibited an increased relative abundance of beneficial bacterial genera, including *Lawsonibacter*, *Acetatifactor*, *Parabacteroides*, *Ruminococcus*, *Bacteroidota*, and *Muribaculum*. These bacteria produce short‐chain fatty acids (SCFAs) that enhance the intestinal barrier, reduce permeability, and exert anti‐inflammatory effects, thus maintaining the stability of the gut microbiome's metabolic processes [67, 68, 69, 70]. Notably, *Muribaculum* may help maintain gut barrier function, preventing the invasion of harmful bacteria and reducing inflammation [71]. Furthermore, studies have shown that enriching the gut microbiome with *Muribaculum* can alleviate symptoms of mental disorders [72, 73]. *Ruminococcus* is known for producing SCFAs, which are associated with the upregulation of tight junction proteins and intestinal mucin expression. These shifts in bacterial abundance suggested a positive therapeutic impact of SP@RIF on the intestinal health of HE mice.

### 2.9 Long‐Term Safety

IEC‐6 cells were used for in vitro experiments. CCK‐8 assays showed that RIF, SP, and SP@RIF had no significant effect on cell proliferation (Figure S14A). Additionally, live/dead staining confirmed that SP@RIF did not harm IEC‐6 cells (Figure S14B). After administering SP@RIF continuously for 30 days, no apparent abnormalities in liver and kidney function were observed in the mice (Figure S14C). Histological examination via H&E staining revealed no significant damage to major organs, including the heart, liver, spleen, lung, and kidney (Figure S14D).

Beyond this oral toxicity assessment, we also evaluated the potential systemic toxicity of RIFnano at the cellular level by testing its cytotoxicity on normal human astrocytes (NHA) and the human hepatocellular carcinoma cell line HepG2. The results demonstrated that RIFnano exhibited minimal cytotoxicity across a range of concentrations (2.5–50 µg/mL) in both cell lines (Figure S15A,C). Furthermore, live/dead staining revealed a predominance of viable cells with negligible dead cells in all treatment groups (Figure S15B,D), further confirming the favorable cytocompatibility of RIFnano. Collectively, these in vitro findings provide direct evidence of the good biocompatibility of RIFnano with astrocytes and hepatocyte‐derived cells, reinforcing the overall biosafety profile of this nanoparticle system alongside the in vivo safety data.

### 2.10 Limitations of the Study

In this study, we demonstrated that SP@RIF may play a role in protecting the intestinal barrier by maintaining its integrity and reducing the absorption of harmful molecules from the gut lumen. Consequently, SP@RIF treatment contributed to lowering blood and brain ammonia levels. We confirmed that SP@RIF treatment modulated the gut microbiota in BDL mice, leading to shifts in bacterial abundance. However, the precise mechanism by which these changes in bacterial composition influence the intestinal barrier or the whole organism remains unclear. Additionally, while SP, as a carrier, independently exerted protective effects on the brain, it is difficult to pinpoint the exact components or pathways responsible for this effect. Further research is needed to elucidate these mechanisms.

## 3 Conclusions

In summary, this study demonstrated that the SP@RIF delivery system holds significant therapeutic potential for the treatment of HE. Compared to free RIF, RIFnano exhibited enhanced antibacterial efficacy in vitro. SP, serving as a functional carrier, not only effectively adsorbed ammonia from the environment but also contributed to the protection of the intestinal barrier. The SP@RIF system enabled sustained release of RIF within the gut, prolonging its local retention and improving bioavailability. This strategy significantly reduced ammonia levels in both the blood and brain, alleviated neuronal damage in the hippocampus, and suppressed neuroinflammation, thus providing superior neuroprotection compared to RIF alone. Moreover, SP@RIF treatment promoted the maintenance of intestinal barrier integrity, as evidenced by improved villus structure and crypt morphology. It also limited the translocation of harmful luminal components such as ammonia into systemic circulation, ultimately contributing to reduced liver injury and improved liver function.

Gut microbiota analysis revealed favorable shifts in microbial composition associated with SP@RIF treatment, suggesting its capacity to modulate the gut environment in HE mice. Importantly, the observed reduction in liver injury, supported by enhanced liver function, reinforces the integrated protective effects of the liver‐gut‐brain axis. The SP@RIF system not only mitigated the harmful effects of ammonia on the brain and gut but also provided substantial protection to the liver, highlighting its potential to address the multi‐organ dysfunction seen in HE. Although behavioral improvements were limited, the comprehensive protective effects on the gut‐liver‐brain axis underscore SP@RIF as a promising therapeutic strategy for HE. This study provides important insights into the interconnected roles of the liver, gut, and brain in HE and warrants further investigation into the mechanisms underlying these effects in future studies.

## 4 Methods

### 4.1 Synthesis and Characterization of SP@RIF

A mixture of 10 mg of RIF (Aladdin) and 100 mg of PLGA (Sigma–Aldrich) was dissolved in 2 mL of dichloromethane (National Pharmaceutical Chemical Reagent Co., Ltd.). Subsequently, a 4 mL solution of 2% polyvinyl alcohol (Shanghai Macklin Biochemical Co., Ltd.) was added to the mixture, which was then homogenized using an ultrasonic probe. The resulting emulsion was stirred overnight at room temperature to allow the evaporation of dichloromethane. Following centrifugation (12 000 rpm for 15 min), the supernatant was discarded, and the RIF@PLGA was collected. A 10 mL solution of 10 mg/mL CS quaternary salt (Shanghai Macklin Biochemical Co., Ltd.) was then added to the RIF@PLGA, and the mixture was stirred at room temperature for 12 h. After centrifugation (12 000 rpm for 15 min), RIFnano was collected. The product was washed three times with pure water to remove any unreacted reagents and then resuspended. The morphology of RIFnano and SP@RIF was examined using SEM and optical microscopy. The size distribution and zeta potential were evaluated using dynamic light scattering with a Malvern Panalytical Zetasizer Nano ZS90. Optical spectra were obtained using a UV–vis–NIR spectrometer (Shimadzu UV‐2600, Japan). A standard curve for RIF was constructed based on the measured absorbance peaks. Various amounts of RIF (2.5, 5, 10, 25, and 50 mg) were added to 100 mg of PLGA to synthesize RIF@PLGA. After washing away the excess RIF, the RIF@PLGA was lysed, and the EE was calculated using the standard curve. 0.6, 0.75, 1, 1.5, and 3 mg of SP were incorporated into 6 mg of RIFnano to synthesize SP@RIF. After lysis of the SP@RIF, the DLE was determined according to the standard curve. To assess the release of RIF, 2 mL of RIFnano suspension was centrifuged, and the precipitate was resuspended in 5 mL of artificial gastric fluid. The mixture was incubated on a shaking bed at 37°C at 50 rpm for 1 h. After centrifugation, the supernatant was discarded, and 5 mL of artificial intestinal fluid was added. The incubation continued for 9 h. At various time points (0.5, 1, 2, 3, 4, 6, 8, and 10 h), 1 mL of the supernatant was sampled to determine the release rate of RIF.

### 4.2 In Vitro Experiments

Cell lines were purchased from EK‐Bioscience, Shanghai. IEC‐6 was cultured in Dulbecco's Modified Eagle Medium, supplemented with 10% fetal bovine serum (FBS) and 1% antibiotics. IEC‐6 cells were seeded in a 96‐well plate (1 × 10^4^ cells per well) and incubated with RIF, SP, and SP@RIF for 24 h, respectively. Normal human astrocytes (NHA) were cultured in astrocyte medium supplemented with 2% fetal FBS and astrocyte growth supplement (ScienCell, USA). The human hepatocellular carcinoma cell line HepG2 was maintained in Dulbecco's Modified Eagle Medium containing 10% FBS, 100 U/mL penicillin, and 100 µg/mL streptomycin. NHA and HepG2 cells were seeded in 96‐well plates at a density of 5 × 10^3^ cells per well and allowed to adhere overnight. The cells were then treated with various concentrations of RIFnano (2.5, 5, 10, 20, and 50 µg/mL) for 24 h. Cell viability was assessed using the Cell Counting Kit‐8 (CCK‐8) assay. The cytocompatibility of RIFnano was further evaluated by live/dead staining using a calcein‐AM/propidium iodide (PI) kit (Invitrogen, USA) according to the kit instructions, followed by imaging with a fluorescence microscope.

*E. coli* were cultured in LB medium and incubated at 37°C, shaking at 200 rpm. The experimental design comprised six groups: LB medium alone (negative control), blank nano, and SP alone as additional controls; and 0.5 mg/mL RIF, 0.5 mg/mL RIFnano, and SP + RIFnano (with 0.5 mg/mL RIF equivalent) as the treatment groups. All groups were incubated overnight. The cultures were then diluted to 10^6^ and plated onto agar plates, with colony counts taken after 24 h. Bacterial staining was performed using N01 and PI according to the kit instructions, and images were captured using a fluorescence microscope. To prepare a series of solutions, 5 mL of 300 µmol/mL ammonium chloride solution was mixed with varying concentrations of SP (0.5, 1, 2, and 4 mg), and the mixture was shaken at room temperature for 12 h. Ammonia concentration in the solution was determined using the indophenol blue method. *E. coli* were each supplemented with an equal volume of LB and incubated with RIF, SP, RIFnano, or SP@RIF overnight at 37°C, shaking at 200 rpm, respectively. Ammonia concentration in the medium was measured using the indophenol blue method.

### 4.3 In Vivo Biodistribution

All animal experiments were approved by the Institutional Animal Care and Use Committee of Zhejiang University. To observe the distribution of the drug in the gastrointestinal tract, male C57BL/6 mice (8 weeks old) were fasted for 12 h. After labeling RIF and SP@RIF with FITC, the mice were orally gavaged, respectively. At 1, 2, 4, 6, and 8 hours post‐gavage, fluorescence imaging of the gastrointestinal tract, heart, liver, spleen, lungs, and kidneys was performed using the PHOTON IMAGER OPTIMA. Fluorescence intensity in the gastrointestinal tract was quantified using ImageJ software (USA). 4 h after gavage, the intestines (duodenum, jejunum, and ileum) and their contents were collected, fixed with 4% paraformaldehyde, sectioned, and stained with DAPI for fluorescence microscopy observation. The intestinal contents were fixed with 2.5% glutaraldehyde solution and examined by SEM.

### 4.4 The Treatment Effect of HE

This study used the TAA and BDL models to simulate HE. The model was performed on male C57BL/6 mice, aged 8 weeks. For the TAA model, mice received intraperitoneal injections of TAA at a dosage of 150 mg/kg body weight once daily for three consecutive days. For the BDL model, the bile duct was separated from the portal vein and hepatic artery, and after freeing the bile duct, it was ligated with 5‐0 silk sutures. The sham surgery group only freed the bile duct without ligating it. Therapeutic interventions commenced 14 days post‐surgery. To evaluate the efficacy of SP@RIF in treating HE, the following treatments were administered once daily: 300 µg of RIF in the RIF group, 200 µg of SP in the SP group, and a combined formulation of SP@RIF (containing 300 µg of RIF and 200 µg of SP) in the SP@RIF group. Mice in the control, sham surgery, TAA, and BDL groups were given an equivalent volume of saline. The body weights of the mice were recorded daily.

### 4.5 Effect on Gut Microbiota

Fecal samples from mice in the sham group and various treatment groups of the BDL model were collected for 16S rRNA gene sequencing to conduct microbiome analysis. The sequencing process included the following steps: DNA extraction, PCR amplification and product purification, fluorescent quantitative analysis of the amplified products, sequencing library preparation, and high‐throughput sequencing.

### 4.6 Biosafety

To evaluate the long‐term safety of SP@RIF, 8‐week‐old male mice were administered SP@RIF (containing 300 µg RIF and 200 µg SP) daily for 30 consecutive days. After one month, the mice were sacrificed, and blood samples along with major organs were collected for hematological and pathological examination.

### 4.7 Statistical Analysis

Differences between groups were analyzed using Student’s *t*‐test or one‐way ANOVA. The statistical significance was indicated as ^*^, *p* < 0.05; ^**^, *p* < 0.01; ^***^, *p* < 0.001; ^****^, *p* < 0.0001; ns, no significance. All statistical analyses were performed using GraphPad Prism v.8.0 (GraphPad Software, USA).

## Author Contributions

K.W., X.H., and L.Y. contributed equally to this work. K.W., X.H., L.Y., J.L., and S.Z. performed experiments. K.W., X.H., and L.Y. completed the data analysis and manuscript writing. M.Z., K.W., and Z.T. designed and supervised the project. All authors have given support to the final version of the manuscript. **Kaiyue Wang**: data curation, formal analysis. **Shaomin Zou**: data curation, formal analysis. **Xueting Huang**: writing – review and editing, methodology, data curation. **Jin Liu**: data curation. **Lingxiao Yang**: methodology, data curation, investigation, formal analysis. **Kai Wang**: conceptualization. **Zhe Tang**: conceptualization, supervision. **Min Zhou**: conceptualization, supervision. **Lei Lei**: data curation.

## Conflicts of Interest

The authors declare no conflicts of interest.

## Supporting information




**Supporting File**: advs76443‐sup‐0001‐SuppMat.docx.

## Data Availability

The data that support the findings of this study are available from the corresponding author upon reasonable request.
