# Supplementary material for: Microalgae Hybrid Biosystem for Enhanced Oral Delivery of Rifaximin in Hepatic Encephalopathy Treatment
Source: Adv Sci (Weinh). 2026 Jul 31;13(54):e76443. doi: 10.1002/advs.76443 (PMC13426099; doi:10.1002/advs.76443)
Supplement: Supplementary file 1 [file ADVS-13-e76443-s001.docx]

**Supporting Information**

**Microalgae Hybrid Biosystem for Enhanced Oral Delivery of Rifaximin in Hepatic Encephalopathy Treatment**

*Kaiyue Wang, Xueting Huang, Lingxiao Yang, Jin Liu, Lei Lei, Shaomin Zou, Kai Wang^*^, Min Zhou^*^, and Zhe Tang^*^*

K. Wang, L. Lei, S. Zou, M. Zhou, Z. Tang

Department of Surgery, the Fourth Affiliated Hospital of School of Medicine, and International School of Medicine, International Institutes of Medicine, Zhejiang University, Yiwu 322000, China

E-mail: zhoum@zju.edu.cn; [8xi@zju.edu.cn](mailto:8xi@zju.edu.cn)

K. Wang

Department of Radiology, Sir Run Run Shaw Hospital, Zhejiang University School of Medicine, Hangzhou 310016, China

X. Huang, M. Zhou

Department of Neurosurgery, the First Affiliated Hospital, School of Medicine, Zhejiang University, Hangzhou 310003, China

L. Yang, K. Wang

Department of Respiratory and Critical Care Medicine, the Fourth Affiliated Hospital of School of Medicine, and International School of Medicine, International Institutes of Medicine, Zhejiang University, Yiwu 322000, China

E-mail: [kaiw@zju.edu.cn](mailto:kaiw@zju.edu.cn)

J. Liu, M. Zhou

Zhejiang University-University of Edinburgh Institute (ZJU-UoE Institute), Zhejiang University School of Medicine, Zhejiang University, Haining 314400, China

Z. Tang

Department of Surgery, The Second Affiliated Hospital, Zhejiang University School of Medicine, Hangzhou 310009, China

**This file includes:**

**Experimental section**

**Supplementary Figures. S1 to S15**

**Experimental section**

Prior to each behavioral test, the mice were allowed to acclimate to the testing environment for at least one hour. The OFT permitted the mice to freely explore a 45 cm × 45 cm × 45 cm area for five minutes. The trajectories of the animals were analyzed, including total distance traveled, distance in the center, and velocity. The tail suspension test involved suspending an isolated mouse 50 cm above the floor, with video recording used to monitor the duration the mouse remained immobile during the last four minutes of the six-minute trial. Liver and brain tissues were homogenized in ice-cold PBS, and protein concentration was measured using a BCA assay kit (Beyotime). Commercial kits (Solarbio) were employed to measure brain ammonia levels, while oxidative stress levels including GSH, MDA and SOD levels in the brain were evaluated using kits from the NanJing JianCheng Bioengineering Institute. ELISA kits (Solarbio) were used to measure the inflammatory cytokines IL-6, IL-1β, and TNF-α in both brain tissue and blood. ALT, AST, TB, DB, BUN, UA, and CR levels in the blood were evaluated using kits from the Nanjing JianCheng Bioengineering Institute.

To assess the histological changes in organs such as the liver, intestine, and brain, a portion of the tissues was fixed overnight in 4% paraformaldehyde at 4°C, embedded in paraffin, and sectioned to obtain 4 μm slices. Nissl staining was performed on the brain sections, while the remaining tissues underwent H&E staining. Additionally, immunofluorescence staining was conducted on the intestinal and brain tissues. A monoclonal antibody against GFAP (Abcam) was used to characterize the astrocyte morphology in the brain, while the Iba-1 antibody (Abcam) was employed to assess microglial morphology. Claudin-1 and ZO-1 proteins were utilized to detect tight junction proteins in the intestine. DAPI was used to stain the cell nuclei. Fluorescence density was quantified using ImageJ software. Furthermore, the hippocampus of the brain was fixed with 2.5% glutaraldehyde solution, sectioned, and examined under TEM.

**Figures**

**Figure S1.** SEM images of nanoparticles stored at 4 °C, room temperature, and 37 °C for 7, 14, and 21 days, respectively.

**Figure S2.** Images of E. coli colonies on LB plates in different groups.

**Figure S3.** Hepatic enzyme levels of mice in different groups (n = 5).

**Figure S4.** Astrocyte staining with GFAP (red) and microglia staining with Iba-1 (green) in the CA1 region of the hippocampus.

**Figure S5.** Microglia (Iba-1 staining, green) in the dentate gyrus (DG) region of the hippocampus and the quantification of Iba1-positive area in the DG region of the hippocampus (n = 3).

**Figure S6.** Levels of inflammatory factors including IFN-γ and CXCL1 in the mouse brain as shown (n = 5).

**Figure S7.** The body weight of the treated mice (n = 10).

**Figure S8.** Serum biochemical tests in treated mice. Results are presented as means ±SD.

**Figure S9.** (A) Representative histological images of the liver in the sham and BDL groups. (B) Hepatic enzyme (ALT, AST) levels in sham and BDL groups (n = 3). (C) Blood ammonia levels in sham and BDL groups (n = 3). (D) TB and DB levels in the sham and BDL group (n = 3).

**Figure S10.** (A) Representative histological images of the brain in the sham and BDL groups. (B) Quantification of brain granular layer thickness (n = 3).

**Figure S11.** Representative tracking plots of mice from the OFT after recovery treatments.

**Figure S12.** Survival curves of mice starting treatment 14 days post-surgery (n = 10).

**Figure S13.** Comparison of relative abundance in *Muribaculum* among groups (n = 5).

**Figure S14.** (A) Cell viability of the IEC-6 cells after incubation RIF, SP and SP@RIF for 24h. (B) Calcein-AM/PI double staining ﬂuorescence images of the IEC-6 cells (green, living cell; red, dead cell). (C and D) Serum biochemical tests (C) and H&E staining images of the major organs(D) of the mice after the daily administration of SP@RIF for 30 days (n = 5).

**Figure S15.** (A) Cell viability of NHA cells treated with RIFnano at different concentrations for 24 h (n = 6). (B) Representative fluorescence microscopy images of live/dead staining in NHA cells after treatment with different concentrations of RIFnano. (C) Cell viability of HepG2 cells treated with RIFnano at different concentrations for 24 h (n = 6). (D) Representative fluorescence microscopy images of live/dead staining in HepG2 cells after treatment with different concentrations of RIFnano.
